# Comparing survival outcomes for cervical cancer based on the 2014 and 2018 International Federation of Gynecology and Obstetrics staging systems

**DOI:** 10.1038/s41598-021-86283-2

**Published:** 2021-03-26

**Authors:** Wonkyo Shin, Tae Young Ham, Young Ran Park, Myong Cheol Lim, Young-Joo Won

**Affiliations:** 1 Center for Gynecologic Cancer, National Cancer Center, Goyang, Korea; 2 Division of Cancer Registration and Surveillance, National Cancer Center, 323 Ilsan-ro, Ilsandong-gu, Goyang, 10408 Korea; 3 Center for Clinical Trials, National Cancer Center, Goyang, Korea; 4 Division of Tumor Immunology, National Cancer Center, Goyang, Korea; 5 Department of Cancer Control and Population Health, Graduate School of Cancer Science and Policy, National Cancer Center, Goyang, Korea

**Keywords:** Cancer, Oncology

## Abstract

The International Federation of Gynecology and Obstetrics (FIGO) cervical cancer staging system was modified in 2018, introducing new stage IB subdivisions and new lymph node status considerations in stage IIIC. We compared cervical cancer survival outcomes according to the 2014 and 2018 FIGO staging systems. We selected 10% of cervical cancer cases (2010–2015) from the Korean national cancer registry (2010–2015) through a systematic sampling method. We collected information using a collaborative stage data collection system and evaluated the results according to both staging systems. The log-rank test was used to analyze overall survival differences. No significant difference in survival was observed between 2018 subdivisions IB1/IB2/IB3 (P = 0.069), whereas a considerable difference was observed between these subdivisions according to histological subtypes. In the 2018 FIGO staging system, stage IIIC had better survival than stage IIIA/IIIB (P < 0.001). We observed considerable heterogeneity in 2018 stage IIIC related to the corresponding stages of the 2014 staging system (stages IA1–IIIB). The size of the primary cervical mass was related to survival (P < 0.001). In conclusion, using lymph node status to define stage IIIC captured a broad range of prognoses. The inclusion of primary tumor size considerations may improve the staging accuracy of advanced cervical cancer.

## Introduction

Clinical staging is a standard approach for classifying patients with cervical cancer^1^, and the extent of local cervical mass spreading is considered one of the most important prognostic factors, relative to lymphatic or hematogenous metastasis. Thus, lymph node status was not included in the staging of cervical cancer, unlike for other gynecologic malignancies, such as ovarian or endometrial cancers^2,3^. After clinical staging, imaging may be used to determine the type of surgery and the need for adjuvant treatment, or it may be used to guide non-surgical treatment using radiotherapy or concurrent chemoradiotherapy. The extent of the primary cervical mass can also vary according to the type of clinical examination, imaging modality, and pathological findings, although this does not lead to re-staging of the tumor. The categorizations in the 2018 International Federation of Gynecology and Obstetrics (FIGO) staging system were changed to reflect the different imaging modalities that are used after clinical examination, which vary according to institution and region. The goal of this modification was to reduce morbidity by accurately staging the disease and guiding appropriate treatment, which can reduce the need for re-operation and radiation therapy^4–7^.

Lymph node status remains a controversial factor in the staging of cervical cancer, although lymph node metastasis is associated with a poor prognosis among patients with the same stage of cervical cancer^8–10^. In addition, a tumor diameter cut-off of 4 cm is used to divide stage IB1/IB2 and stage IIA1/IIA2 in 2014 FIGO system, and there is also a need to consider further subdividing tumors with a diameter of < 4 cm^11,12^. The 2014 FIGO stage IB1 classification was subsequently divided into IB1 and IB2 in the 2018 FIGO system, while the 2014 stage IB2 was re-defined as stage IB3 in the 2018 system. Furthermore, the 2018 FIGO system considered lymph node status for stage IIIC disease, with pelvic lymph node metastasis categorized as stage IIIC1 and para-aortic lymph node metastasis categorized as stage IIIC2 (regardless of primary tumor size), based on pathological or radiological results^7^ (Supplementary Table 1).

Matsuo et al. performed a validation study of the 2018 FIGO staging system using the Surveillance, Epidemiology, and End Results data^13^. Interestingly, a significant difference was observed between the stage IB1/IB2 subdivisions, with a somewhat meaningful difference in the histological characteristics of the stage IB1/IB2/IB3 classifications (e.g., adenocarcinoma was more common in stage IB1 and squamous cell carcinoma was most common in stage IB3). Furthermore, the survival curves for stage IIIC1 were superior to those for stage IIIA/IIIB, which was attributed to the heterogeneity of stage IIIC1 cases^13^. However, the Surveillance, Epidemiology, and End Results data did not include para-aortic lymph node status, which precluded an analysis of stage IIIC2, and there were limited data regarding the radiological or pathological findings for lymph node status. Therefore, the present study aimed to compare survival outcomes between the 2014 and 2018 FIGO systems, to determine the utility of the new 2018 FIGO staging system, using data from the Korean national cancer registry.

## Results

**Figure 1:**
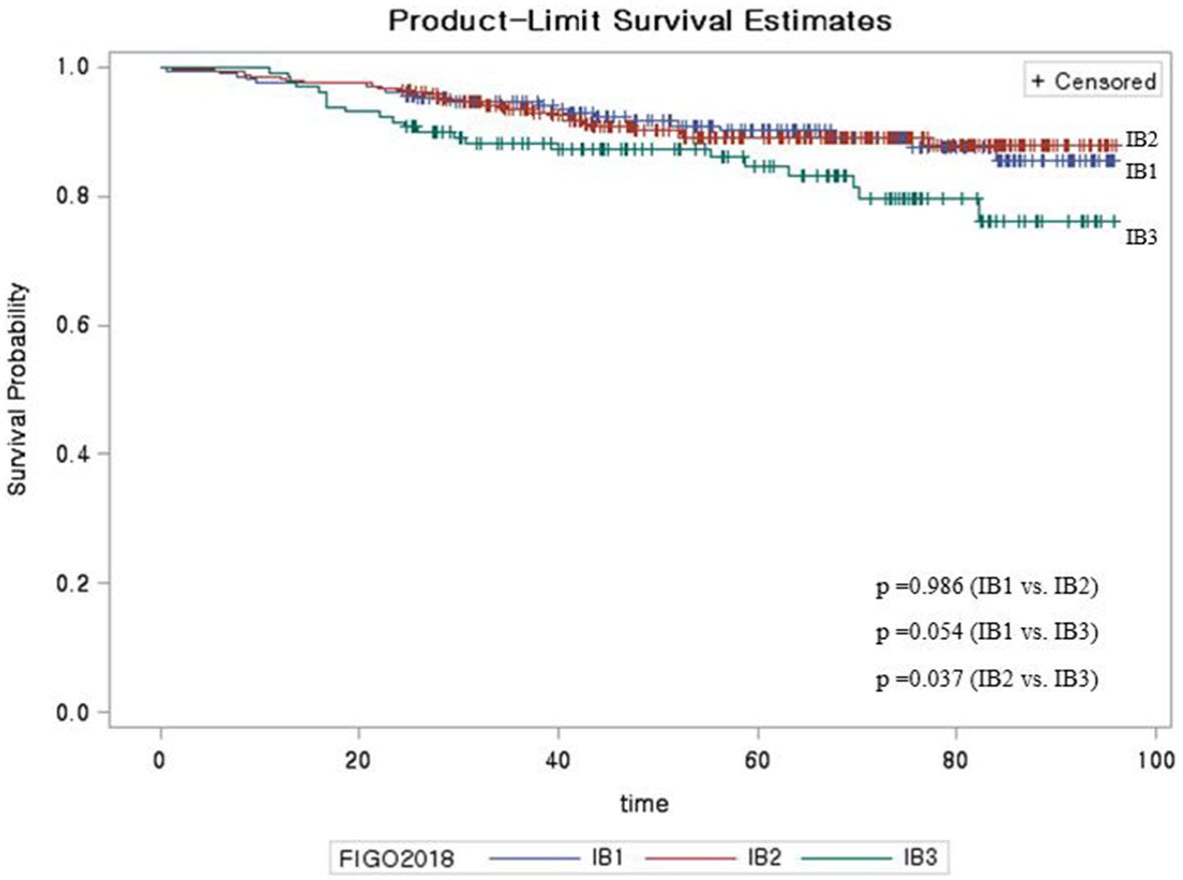
Kaplan–Meier analysis of survival for 2018 FIGO stages IB1/IB2/IB3.

A total of 2441 patients with cervical cancer were included in this study and their baseline characteristics are shown in Table 1. Figure 1 shows the survival curves for 2018 FIGO stage IB subgroups (except for stage unknown). A small but non-significant difference was observed between the survival curves for 2018 FIGO stage IB1/IB2/IB3 subdivisions (P = 0.069). We also evaluated the histological classifications for stage IB subgroups, which revealed that squamous cell carcinoma was more common in stage IB3 than that in stage IB1 (74.6% vs. 70.3%), whereas adenocarcinoma was more common in stage IB1 than that in stage IB3 (25.4% vs. 18.5%). Intermediate results were observed for stage IB2. Adenosquamous carcinoma accounted for the smallest proportion of cases in the subgroups (Table 2).

**Table 1:** Baseline characteristics of Korean patients with cervical cancer.

Category	All patients N = 2441 n (%)
**Age (years)**	
< 40	438 (17.9)
40–49	632 (25.9)
50–59	570 (23.4)
60–69	353 (14.5)
≥ 70	448 (18.4)
**Year at diagnosis**	
2010	443 (18.2)
2011	407 (16.7)
2012	404 (16.6)
2013	407 (16.7)
2014	396 (16.2)
2015	384 (15.7)
**Histological subtype**	
Squamous cell carcinoma	1897 (77.7)
Adenocarcinoma	379 (15.5)
Adenosquamous carcinoma	62 (2.5)
Others	103 (4.2)
**AJCC 7th edition, T status**	
T1	1415 (58.0)
T2	663 (27.2)
T3	174 (7.1)
T4	52 (2.1)
TX	113 (4.6)
Tis	24 (1.0)
**2018 FIGO stage**	
I	1259 (51.6)
II	383 (15.7)
III	502 (20.6)
IV	188 (7.7)
Unknown/Tis	109 (4.5)
**2014 FIGO stage**	
I	1395 (57.2)
II	615 (25.2)
III	133 (5.5)
IV	181 (7.4)
Unknown/Tis	117 (4.8)

*FIGO* International Federation of Gynecology and Obstetrics, *AJCC* American Joint Committee on Cancer.

**Table 2:** The histological distributions of stage IB1/IB2/IB3 based on the 2018 FIGO stage.

Histological subtype	2018 FIGO						
	IB1		IB2		IB3		P value
	n	% (95% CI)	n	% (95% CI)	n	% (95% CI)	
Squamous cell carcinoma	147	70.3 (64.11–76.49)	194	68.6 (63.19–74.01)	97	74.6 (67.12–82.08)	0.1928
Adenocarcinoma	53	25.4 (19.5–31.3)	74	26.2 (21.08–31.32)	24	18.5 (11.83–25.17)	0.0645
Adenosquamous carcinoma	6	2.9 (0.62–5.18)	8	2.8 (0.88–4.72)	4	3.1 (0.12–6.08)	0.4584
Others	3	1.4 (–0.19–2.99)	7	2.5 (0.68–4.32)	5	3.9 (0.57–7.23)	0.0921

*FIGO* International Federation of Gynecology and Obstetrics.

**Figure 2:**
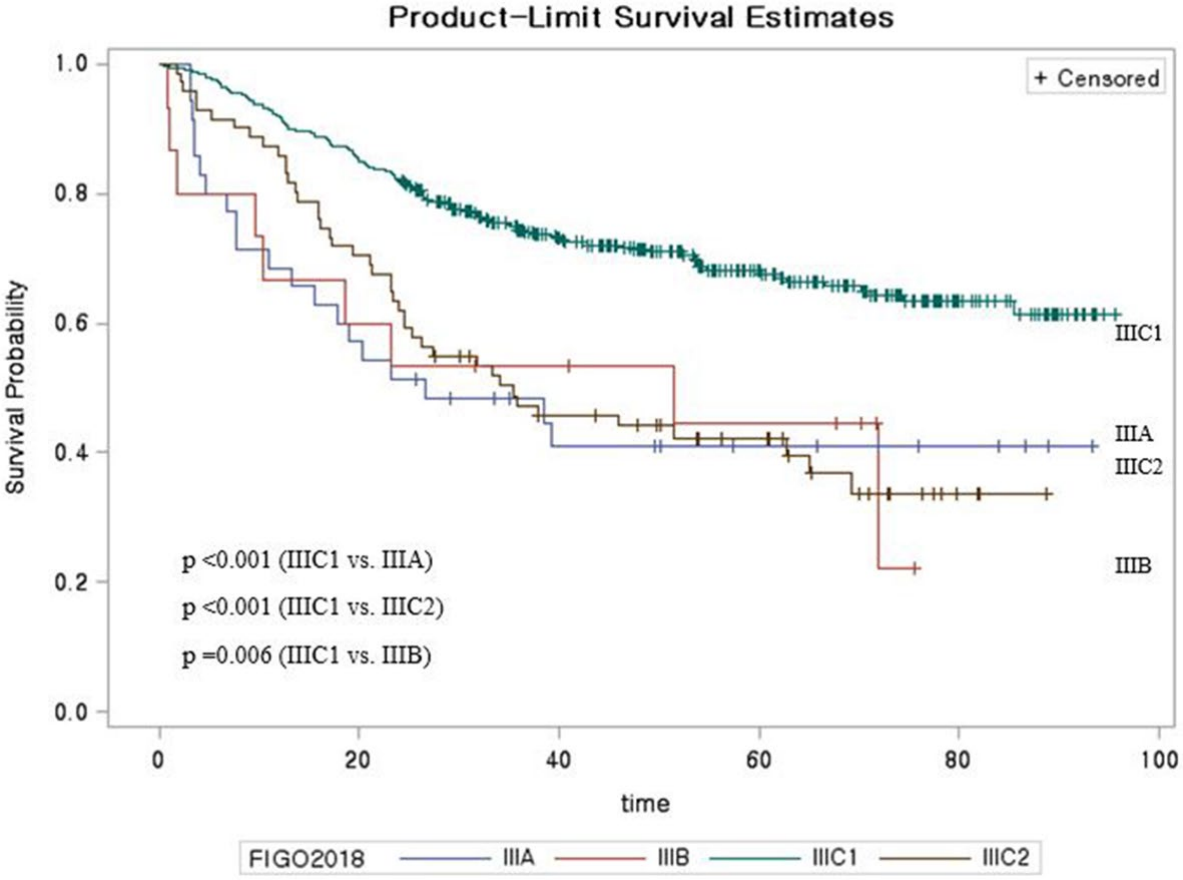
Kaplan–Meier analysis of survival for 2018 FIGO stage III.

**Figure 3:**
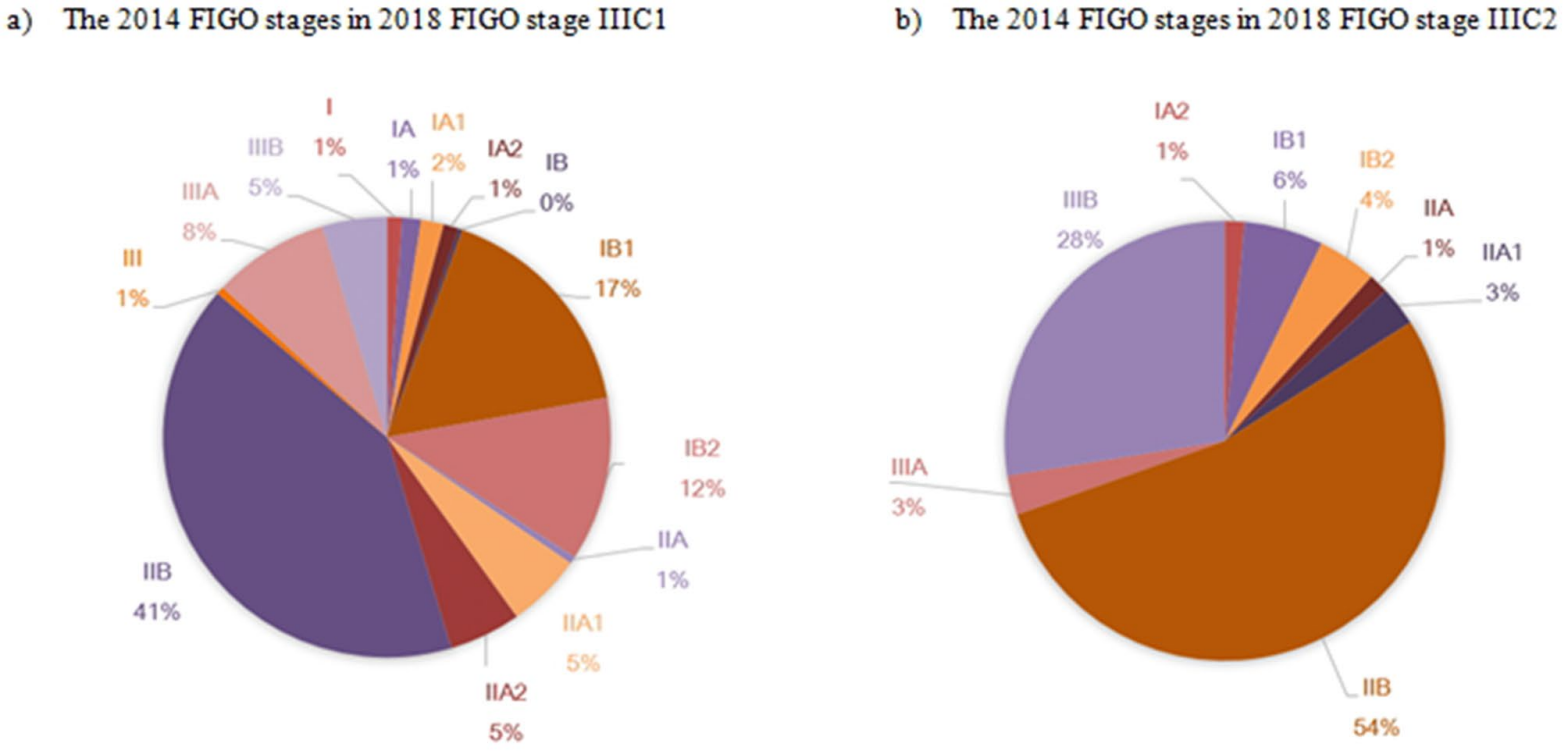
Distribution of 2014 FIGO stages in the 2018 FIGO stages IIIC1 (**a**) and IIIC2 (**b**).

We also analyzed data for the newly added 2018 FIGO stage IIIC, with pelvic lymph node metastasis classified as stage IIIC1 and para-aortic lymph node metastasis classified as stage IIIC2, based on either radiological or pathological confirmation. It is important to note that patients with stage IIIC from the 2018 FIGO system would have been assigned to other groups based on the 2014 FIGO system. In the 2018 FIGO system, stage IIIC1/IIIC2 had better survival than stage IIIA/IIIB, and stage IIIC1 had better survival than stage IIIC2 (P < 0.001) (Fig. 2). Patients in the stage IIIC group from the 2018 FIGO system were most commonly assigned to stage IIB in the 2014 FIGO system, although the 2014 stages ranged broadly from IA1 to IIIB (Fig. 3). We also sub-classified the stage IIIC group according to the T classification of the primary cervical mass and observed significant differences in the survival curves for T1, T2, and T3 (P < 0.001) (Supplementary Figure S1).

## Discussion

Previous reports have suggested that the 2014 FIGO stage IB1 group should be further subdivided^12,14,15^. Furthermore, in 2018, the results of the LAAC trial (locally advanced cervical cancer)^11^ reported that laparoscopic surgery is more dangerous than laparotomy for locally advanced cervical cancer. However, many reports have suggested that laparoscopic surgery was not dangerous if the cervical mass was small^11,15,16^. Thus, the division of stage IB1 from the 2014 FIGO system into stage IB1/IB2 from the 2018 FIGO system may help better guide patient management. In addition, the 2018 European Society of Gynecological Oncology/European Society for Radiotherapy and Oncology/European Society of Pathology guidelines suggested that immediate concurrent chemoradiotherapy might be considered without surgery if the cervical mass was > 2 cm, rather than > 4 cm^12^. Thus, the new 2018 staging system may be useful. Although the present study failed to detect a significant difference in survival between the new stages IB1 and IB2 (P = 0.069), a significant difference was detected in the analysis of Surveillance, Epidemiology, and End Results data from the US (P < 0.001)^13^. This discrepancy may be related to the difference in the sample sizes of stage IB cases between the two studies (approximately 8000 patients from the Surveillance, Epidemiology, and End Results database versus only approximately 1000 patients in the present study).

Furthermore, according to the FIGO 2018 staging system, if the LN metastasis is radiologically or pathologically confirmed, it is categorized as stage IIIC, not stage I. Notably, heterogeneity in stage I did not occur due to other prognostic factors, since it was analyzed only with the size of the original cervix mass.

The radicality of hysterectomy is also generally different for cases involving stage IB1 or IB2 disease. However, the Surveillance, Epidemiology, and End Results data analysis only considered simple or extended hysterectomy, while the present study was unable to determine the extent of surgery, which may have affected our findings. There are several criteria to consider when identifying hysterectomy types in patients with cervical cancer, and the most widely used are those associated with the Querleu–Morrow classification^17,18^. The surgical factors are very important in determining the subsequent prognosis, which suggests that a detailed analysis of surgical factors is needed to confirm the survival difference between stage IB1 and IB2 cases.

The incidence of AC is relatively high in IB1, and the incidence of SCC is high in IB3. In this aspect, it is necessary to consider that SCC and AC have different pathophysiology, which includes epidemiology, tumor spreading pattern, and prognosis^19–22^. SCC typically spreads locally and invades adjacent organs, whereas AC more frequently exhibits a lymphatic or hematogenous tumor cell spreading pattern. Therefore, as the size of the primary site increases, the probability of AC spreading to other organs increases. Consequently, it is possible for a patient to be diagnosed as a stage III or higher, but not IB3.

The new stage IIIC criteria consider lymph node status based on clinical, radiological, and pathological findings. Interestingly, the present study revealed that stage IIIC1/IIIC2 had better survival than stage IIIA/IIIB. Further analysis of the stage IIIC group from the 2018 FIGO system revealed broad differences in staging according to the 2014 FIGO system (ranging from stages IA1 to IIIB) (Fig. 3). Traditionally, the FIGO staging of cervical cancer has been determined by the size and adjacent invasion extent of the primary cervical mass. The 2018 system only considers lymph node status for stage IIIC and omits the cervical tumor’s extent and invasion status. This definition of stage IIIC may lead to better survival outcomes for stage IIIC, relative to stage IIIA/IIIB. We evaluated this issue by considering survival in stage IIIC cases according to T classification (Supplementary Figure S1), which revealed significant survival differences according to T classification. Therefore, it appears that only considering lymph node status is insufficient for determining the clinical characteristics of stage IIIC cervical cancer and may not be appropriate in a clinical setting, given the heterogeneity in this patient group. Lymph node metastasis is classified as an isolated tumor cells (if LN size < 0.2 mm), micrometastasis (0.2 mm < LN < 2 mm), and macrometastasis (> 2 mm). For isolated tumor cells, it is recorded in the pathologic report, but it is not classified as stage IIIC. Micrometastasis is also regarded as macrometastasis. Although, our data do not have the detailed LN status; comparing the survival according to the LN metastatic status could show interesting results^23^.

By adding lymph node status, the difference between 2018 FIGO staging and TNM staging has been reduced, although the 2018 FIGO system still creates a heterogeneous stage IIIC group. Thus, additional information regarding the primary cervical mass is needed to reduce the heterogeneity in stage IIIC. The standard treatments for locally advanced cervical cancer can involve radical hysterectomy, radiotherapy, or concurrent chemoradiotherapy. While various criteria are used to select the appropriate treatment, radical hysterectomy is generally performed for cases involving stage IIB or lower, while concurrent chemoradiotherapy is recommended for more advanced cases^1,24^. Some patients with early-stage disease have had their lymph node status diagnosed based on imaging, which may help guide the use of adjuvant treatment when metastasis in confirmed via lymphadenectomy and during surgery. Within the same disease stage, lymph node metastasis appears to be associated with a poor prognosis, although this does not appear to outweigh the prognostic relevance of the primary cervical mass status^8^. For example, based on the 2018 FIGO system, surgical treatment might be selected for patients with lymph node metastasis that is identified via imaging (i.e., stage IIIC), regardless of the primary tumor’s status, while other patients might receive concurrent chemoradiotherapy based on the tumor’s size and adjacent organ invasion, even if they were also diagnosed with stage IIIC disease. This may cause issues in the real-world management of these patients.

In conclusion, this retrospective study surveyed data from the Korean national cancer registry and classified the cases according to the 2014 and 2018 FIGO systems. The effectiveness of the stage IB subdivisions in the 2018 FIGO system was not confirmed, which may have been related to the relatively small sample size. Furthermore, stage IIIC disease had a better prognosis than stage IIIA/IIIB disease based on the 2018 FIGO staging system, and there was substantial heterogeneity among stage IIIC cases. Additional discussion is needed to address how lymph node status should be managed in the FIGO staging system for Korean patients with cervical cancer.

## Methods

### Study population and methods

This study evaluated data from patients who were registered in the Korean Central Cancer Registry, which is a nationwide cancer registry that includes > 95% of Korean cancer cases. For the present study, a random sample was selected from the Korean Central Cancer Registry to obtain additional data regarding 10% of patients diagnosed with cervical cancer between 2010 and 2015. The study’s retrospective protocol was reviewed and approved by the institutional review board of the National Cancer Center (approval number: NCC2019-0060), and the requirement for informed consent was waived. All study methods complied with the applicable national guidelines and regulations regarding the use of registry data.

A standardized protocol was used for a collaborative stage data collection system^25^, which was developed by the American Joint Committee on Cancer to collect basic information required for cancer staging. This system was used to collect data regarding prognostic factors required for stage grouping (tumor size, lymph node status, and metastasis at diagnosis) and additional recommended factors (pelvic nodal status, para-aortic nodal status, mediastinal nodal status, and scalene nodal status).

The sampled survey database uses variables that are similar to those used by the Surveillance, Epidemiology, and End Results database rather than being based on the FIGO system. After the data were sorted according to the TNM criteria, the cases were classified according to the 2014 and 2018 FIGO staging systems. Patients were followed until December 31, 2017, and survival outcomes were compared between the various stages that were determined using the 2014 and 2018 FIGO staging systems.

### Statistical analysis

Clinical characteristics were grouped as categorical variables and data were reported as number and percentage. The Kaplan–Meier method was used to create overall survival curves for the various stage groups, and the curves were compared using the log-rank test. Differences were considered statistically significant at P values < 0.05. All analyses were performed using SAS software (version 9.4; SAS Institute Inc., Cary, NC, USA).

## Data availability

All data generated or analyzed during this study are included in this published article (and its Supplementary Information files).

## Supplementary Information

Supplementary Information.
