# Supplementary material for: Comparing survival outcomes for cervical cancer based on the 2014 and 2018 International Federation of Gynecology and Obstetrics staging systems
Source: Sci Rep. 2021 Mar 26;11:6988. doi: 10.1038/s41598-021-86283-2 (PMC7997868; doi:10.1038/s41598-021-86283-2)
Supplement: Supplementary file 1 — Supplementary Information. [file 41598_2021_86283_MOESM1_ESM.docx]

**Comparing survival outcomes for cervical cancer based on the 2014 and 2018 International Federation of Gynecology and Obstetrics staging systems**

Wonkyo Shin, MD, PhD ^1^, Tae Young Ham, BS ^2^, Young Ran Park, MS ^2^, Myong Cheol Lim, MD, PhD ^1,3,4,5^, Young-Joo Won, MD, PhD ^2,5*^

^1^ Center for Gynecologic Cancer, National Cancer Center, Goyang, Korea

^2^ Division of Cancer Registration and Surveillance, National Cancer Center, Goyang, Korea

^3^ Center for Clinical Trials, National Cancer Center, Goyang, Korea

^4^ Division of Tumor Immunology, National Cancer Center, Goyang, Korea

^5^ Department of Cancer Control & Population Health, Graduate School of Cancer Science and Policy, National Cancer Center, Goyang, Korea

***Correspondence to**: Young-Joo Won, PhD

Division of Cancer Registration and Surveillance, National Cancer Center

323 Ilsan-ro, Ilsandong-gu, Goyang-si, 10408, Korea

E-mail: [astra67@ncc.re.kr](mailto:astra67@ncc.re.kr)

**Supplementary Figure S1.** The survival curves for stage IIIC according to T classification

(p<0.001)


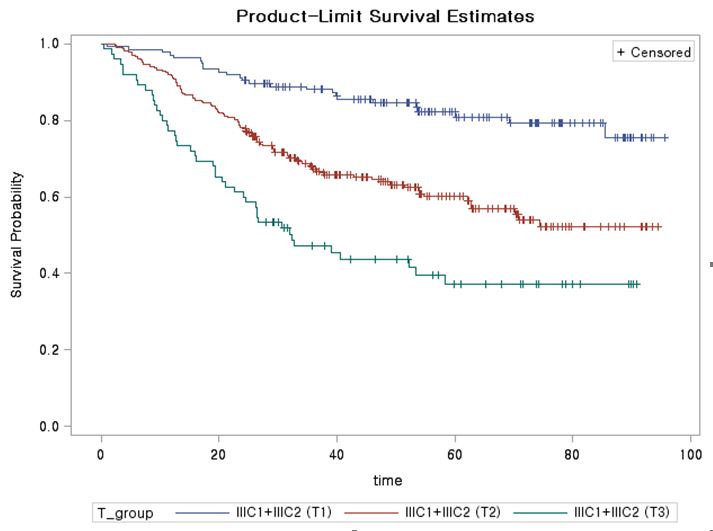


**Supplementary table 1. Changes in cervical cancer staging between 2014 and 2018**

| Stage | FIGO 2014 |  | FIGO 2018 |
| --- | --- | --- | --- |
| IB | Clinical lesions confined to the cervix, or preclinical lesions greater than stage IA. |  | Invasive carcinoma with measured deepest invasion ≥5 mm (greater than Stage IA), lesion limited to the cervix uteri |
| IB1 | Clinical lesions no greater than 4 cm in size. |  | Invasive carcinoma ≥5 mm depth of stromal invasion, and <2 cm in greatest dimension |
| IB2 | Clinical lesions > 4 cm in size. |  | Invasive carcinoma ≥2 cm and <4 cm in greatest dimension |
| IB3 | N/A |  | Invasive carcinoma ≥4 cm in greatest dimension |
| IIIC | N/A |  | Involvement of pelvic and/or para-aortic lymph nodes, irrespective of tumor size and extent (with r and p notations) |
| IIIC1 | N/A |  | Pelvic lymph node metastasis only |
| IIIC2 | N/A |  | Para-aortic lymph node metastasis |
